# Associating the scale-up of insecticide-treated nets and use with the decline in all-cause child mortality in the Democratic Republic of Congo from 2005 to 2014

**DOI:** 10.1186/s12936-021-03771-6

**Published:** 2021-05-29

**Authors:** Johanna Karemere, Ismael G. Nana, Andrew Andrada, Olivier Kakesa, Eric Mukomena Sompwe, Joris Likwela Losimba, Jacques Emina, Aboubacar Sadou, Michael Humes, Yazoumé Yé

**Affiliations:** 1 PMI Measure Malaria, University of North Carolina at Chapel Hill, Chapel Hill, NC USA; 2 ICF, Rockville, MD USA; 3 National Malaria Control Programme, Ministry of Health, Kinshasa, Democratic Republic of Congo; 4 University of Lubumbashi, Lubumbashi, Democratic Republic of Congo; 5 University of Kisangani, Kisangani, Democratic Republic of Congo; 6 University of Kinshasa, Kinshasa, Democratic Republic of Congo; 7 Population and Health Research Institute, Kinshasa, Democratic Republic of Congo; 8 President’s Malaria Initiative/U.S. Agency for International Development, Washington, DC USA

**Keywords:** Malaria, Control, ITN, Kaplan–Meier, Cox proportional hazards, All-cause child mortality, Sub-Saharan Africa, DRC

## Abstract

**Background:**

To reduce the malaria burden and improve the socioeconomic status of its citizens, the Democratic Republic of Congo scaled up key malaria control interventions, especially insecticide-treated nets (ITNs), between 2005 and 2014. Since then, the effects of these interventions on malaria mortality and morbidity have not been assessed. This study aimed to measure the impact of the National Malaria Control Programme’s efforts and to inform future control strategies.

**Methods:**

The authors used data from the Demographic and Health Surveys 2007 and 2013–2014 to assess trends in all-cause childhood mortality (ACCM) against trends in coverage of malaria interventions at national and subnational levels. The authors used the plausibility argument to assess the impact of the malaria control interventions and used Kaplan–Meier survival probability and Cox proportional hazard models to examine the effect of ITN ownership on child survival. Contextual factor trends affecting child survival were also considered.

**Results:**

Countrywide, household ownership of at least one ITN increased, from 9% in 2007 to 70% in 2013–2014. All provinces experienced similar increases, with some greater than the national level. ITN use increased between 2007 and 2013–2014 among children under five (6% to 55%). Severe anaemia (haemoglobin concentration < 8 g/dl) prevalence among children aged 6–59 months significantly decreased, from 11% (95% confidence interval [CI] 9–13%) in 2007 to 6% (95% CI 5–7%) in 2013–2014. During the same period, ACCM declined, from 148 (95% CI 132–163) to 104 (95% CI 97–112) deaths per 1000 live births. The decline in ACCM was greater among children aged 6–23 months (relative reduction of 36%), compared to children aged 24–59 months (relative reduction of 12%). Cox regression indicated that household ownership of at least one ITN reduced the risk of mortality by 24% among children under five (risk ratio = 0.76, 95% CI 0.64–0.90). Contextual factor analysis revealed marginal improvements in socioeconomic indicators and other health interventions.

**Conclusions:**

Given the patterns of the coverage of malaria control interventions, patterns in ACCM by province, and marginal improvements in contextual factors, the authors conclude that the malaria control interventions have plausibly contributed to the decrease in ACCM in the Democratic Republic of Congo from 2005 to 2014.

## Background

**Fig. 1:**
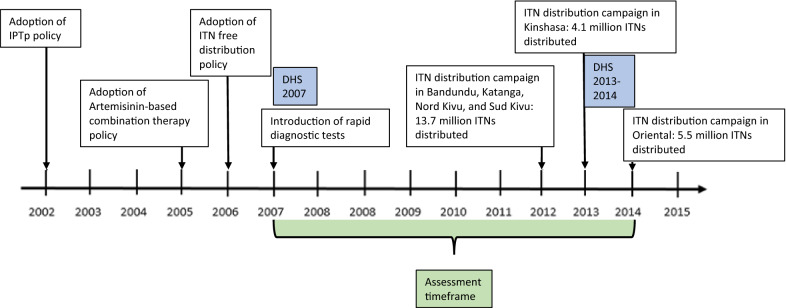
Intervention policy and implementation timeline. *IPTp* intermittent preventive treatment in pregnancy, *ITN* insecticide-treated net, *DHS* demographic and health survey

Over the last decade, malaria has been the leading cause of mortality and morbidity in the Democratic Republic of Congo (DRC), with an estimated 97% of the Congolese population at risk of malaria infection [1, 2]. To improve the overall health of its citizens and reduce the socioeconomic burden of malaria, the country and its technical and financial partners invested significantly in proven malaria control interventions between 2005 and 2015. Using these funds, the National Malaria Control Programme (NMCP) set objectives to contribute to the elimination of malaria and reduce the malaria mortality and morbidity rates by 50% compared to the 2010 levels by 2015 [3]. The NMCP’s strategy was to scale up and expand access to these key interventions: the use of insecticide-treated nets (ITNs), intermittent preventive treatment in pregnancy (IPTp), indoor residual spraying (IRS), and prompt and effective malaria case management [3, 4]. DRC’s efforts to achieve its malaria control goals focused on the scale-up of ITN coverage through free ITN mass distribution campaigns and routine distribution during antenatal care visits and expanded programme on immunization (EPI) visits (Fig. 1) [3].

The effects of scale up of malaria control intervention on all-cause child mortality (ACCM) have been studied in other countries in sub-Saharan Africa, however, no studies have been conducted in DRC [5, 6]. This study aimed to fill this gap by using Demographic and Health Survey (DHS) data to assess the extent to which malaria control interventions have been implemented and scaled up, assess the trends of malaria morbidity and mortality from 2005 to 2014, and evaluate plausible association between interventions and the trends of the disease burden. The current national malaria strategic plan (NMSP) encompasses the period from 2016 to 2020, and the latest available DHS survey is from 2013 to 2014, which precludes the assessment of more recent trends [2, 7]. The results of this assessment allows for a comparison between the intervention coverage achieved and the NMSP targets set for 2015 [3]. In addition, the results will be a key resource in providing baseline data on intervention coverage and malaria morbidity and mortality for the current 2016–2020 NMSP. Following the end of this NMSP, another evaluation can then be used to compare this baseline and evaluate the NMCP progress made on these key indicators [7].

## Methods

### Data sources

This evaluation used nationally representative household survey data from the 2007 and 2013–2014 DHS [1, 2]. The authors omitted the 2010 and 2017–2018 DRC Multiple Indicator Cluster Survey from this study because of incongruous mortality estimate methodology. DHS calculates child mortality based on the birth history of women of childbearing age compared to the MICS, which calculates child mortality indirectly using the number of children born alive. Differences in sampling strategies may also affect interpretation of trends. An exploratory analysis of malaria indicators by region showed different trends across the surveys. In most cases, the MICS estimates were higher compared to the DHS, suggesting a sampling difference. The contextual factor data were obtained from the DHS. Gross domestic product per capita purchasing power parity, total annual rainfall, and temperature data were from the World Bank [8, 9].

### Study design

This evaluation used the before-and-after plausibility approach as recommended by the Roll Back Malaria Partnership to End Malaria Monitoring and Evaluation Reference Group [10]. The rationale and use of this evaluation design to measure the primary impact of malaria control intervention scale-up on ACCM has been detailed in elsewhere [5, 11–13]. Following previous research on the effectiveness of malaria control interventions, the authors hypothesized that improvement in coverage of these interventions (ITNs, IRS, IPTp, and case management) should result in a reduction in ACCM, assuming that other contextual factors (e.g., living conditions, nutritional status of children) have not substantially changed over the evaluation period [5]. The plausibility approach is strengthened by the simultaneous decline in other outcome measures such as malaria prevalence and severe anaemia concurrent with malaria control intervention scale-up. The use of ACCM as the primary impact indicator provides a robust measure that accounts for direct and indirect malaria mortality. In addition to the mortality indicator, morbidity was assessed through measuring trends in severe anaemia [haemoglobin (Hb) concentration < 8 g/dl] prevalence among children aged 6–59 months from 2005 to 2013–2014 [14]. Data on the prevalence of malaria among children aged 6–59 months were not available for the DHS 2007; therefore, it was not possible to assess malaria infection trends during the evaluation period.

### Analytical approach

#### Trends analysis

Descriptive analysis was conducted using Stata version 14 (Stata Corporation, College Station, TX). To account for the hierarchical design, the SVY commands were used, with household weights considered for each analysis. Percentages and rates were calculated with 95% confidence intervals (CIs) to assess trends between 2007 and 2013–2014 at the national and subnational levels. The 11 provinces that constituted DRC until 2015 were examined. The analysis encompasses changes in intervention coverage, including household ownership and use of ITNs and access to malaria diagnosis and treatment for children with fever, but also changes in outcome measures such as severe anaemia and ACCM. Key household survey indicators used for this evaluation are provided in Table 1 [15].

**Table 1:** Key household surveys indicators used for this evaluation [15]

Indicator	Definition
Vector control indicators	
ITNs	Proportion of households with at least one ITN
	Proportion of households with at least one ITN for every two people
	Proportion of children under 5 years old who slept under an ITN the previous night
Case management indicators	
Early access to diagnosis and effective treatment	Proportion of children under 5 years old with fever in last 2 weeks who had a finger or heel stick
	Proportion receiving first-line treatment, among children under 5 years old with fever in the last 2 weeks
Impact indicators	
Morbidity	Proportion of children aged 6–59 months with a haemoglobin measurement of < 8 g/dl
Mortality	All-cause mortality in children under five (5q0)

#### Regression analysis

The regression analysis included Kaplan–Meier survival and Cox proportional hazard models to support the plausibility argument. The Kaplan–Meier survival analysis used the full birth history data from the DHS 2013–2014, which were transformed into a 10-year retrospective (2004–2013) longitudinal dataset reflecting individual child observations from birth until the date of the survey or, in the unfortunate event, the death of the child. Kaplan–Meier survival estimates were calculated, and comparisons were made for survival probability of children aged 0–59 months before (2004–2008) and after (2009–2013) the expansion of malaria control interventions. The assumption is that child survival would improve post-intervention scale-up, compared to pre-intervention scale-up. The outcome variable was defined as the age at which a child dies or the age at interview for those who survived. A dichotomous variable (coded 1 if the child died and 0 if the child was alive) was used to define the censoring status.

Cox proportional hazards regression used the same longitudinal birth history dataset as the Kaplan–Meier analysis. This model allows for use of time-varying covariates to produce a hazard ratio [16–18]. When the hazard ratio is greater than one, there is an increased risk of mortality in the corresponding category compared to the reference category. Conversely, the risk of dying is lower when the hazard ratio is less than one. The hazard rate in the Cox model is computed as:

$$h\left( \frac{t}{zj} \right) = h_{0 } \left( t \right),exp\left( {\beta j\;zj\left( t \right)} \right),$$

where the regression coefficients are to be estimated from the data. The term $h_{0 }$(t) is the baseline hazard function (the hazard when z = 0), zj(t) is the individual covariates vector, and $\beta j$ is a vector of the regression parameters that indicates the effects of these covariates, some of them varying with t (hence, the term time-varying covariate). The relative hazards are given by $exp(\beta j zj\left( t \right).$

To identify an individual child’s exposure to an ITN, data on the duration of ownership of ITNs were used to construct a time-varying variable of ITN ownership for up to 2 years before the survey (2011–2013). The model included all children aged 0–59 months for the 2-year period of exposure to ITN ownership to account for recall bias related to duration of ITN ownership. The analysis time was defined in months from the beginning of the period, allowing the introduction of age as a covariate in the analysis. Each child was observed from the beginning of the observation period until censoring due to death or until the date of the survey. A dichotomous variable (coded 1 if the child died and 0 if the child was alive) was used to define the censoring status. The Cox proportional hazards model assessed the relationship between household ITN ownership and child mortality (deaths of children aged 0–59 months) over the 24 months preceding the survey in each malaria-endemic zone. The model was adjusted for child’s age (month), child’s sex, mother’s age (year) at child’s birth, mother’s education, parity (number of children ever born), household wealth quintiles, and place of residence (urban/rural) because these co-variates are likely to be associated with both mortality and household ownership of ITN.

## Results

### Trend in intervention coverage

#### Ownership of ITNs

**Fig. 2:**
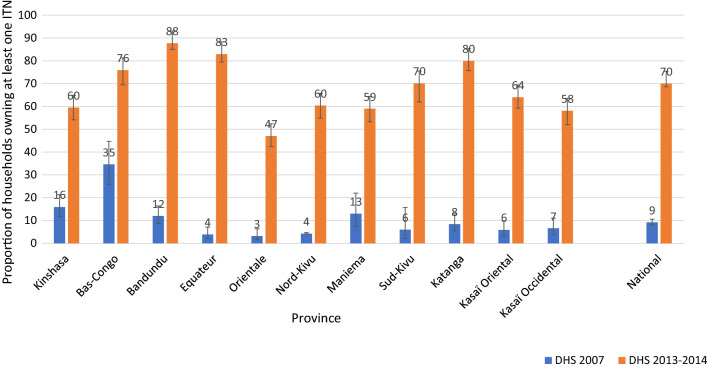
Evolution of the proportion of households with at least one ITN at the national level and by province, in the DRC from 2007 to 2013. *ITN* insecticide-treated net

At the national level, the proportion of households owning at least one ITN increased significantly by 61 percentage points, from 9% (95% CI 8.0–10.6%) in 2007 to 70% (95% CI 68.6–71.4%) in 2013. Similar patterns were observed in all the provinces, with percentage point increases higher than the national increase in Equateur (79 percentage points), Bandundu (76 percentage points), Katanga (72 percentage points), and Sud-Kivu (64 percentage points) (Fig. 2).

#### Use of ITNs among children under five

**Fig. 3:**
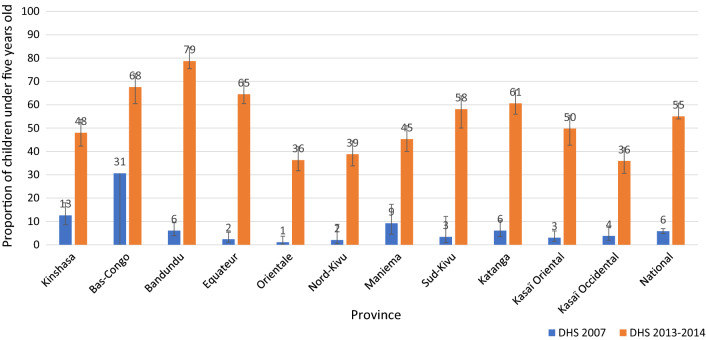
Evolution of proportion of children under 5 years old who slept under an ITN the previous night at the national level and by province, in the DRC from 2007 to 2013. *ITN* insecticide-treated net

At the national level, the use of ITNs in children under 5 years of age in all households significantly increased, from 6% (95% CI 5–7%) in 2007 to 55% (95% CI 54–57%) in 2013. The highest increases were observed in the provinces of Bandundu (73 percentage points), Equateur (63 percentage points), Katanga (55 percentage points), and Sud-Kivu (55 percentage points) (Fig. 3).

#### Use of recommended malaria treatment among children under five

**Fig. 4:**
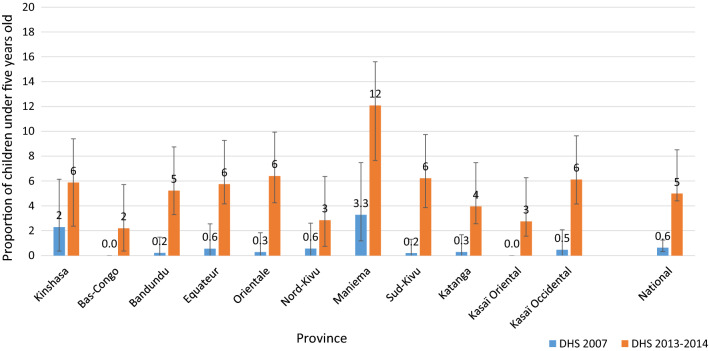
Evolution of the proportion of children under five with fever treated with an ACT the national level and by province, in the DRC from 2007 to 2013. *ACT* artemisinin-based combination therapy

The proportion of children under five with fever in the 2 weeks preceding the survey treated with artemisinin-based combination therapy (ACT) slightly improved from 2007, with an increase from less than 1% in 2007 to 5% in 2013 (Fig. 4). Subnationally, the use of ACT also increased, with improvements in every province. However, access to ACT remained very low, with 5% of children with fever treated with ACT nationally.

### Change in morbidity and mortality

#### Morbidity: change in severe anaemia

**Fig. 5:**
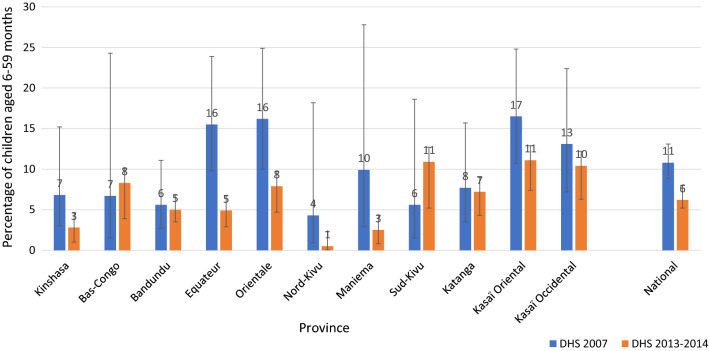
Prevalence of severe anaemia (Hb < 8 g/dl) among children aged 6–59 months by province, in the DRC from 2007 to 2013. *Hb* haemoglobin

**Fig. 6:**
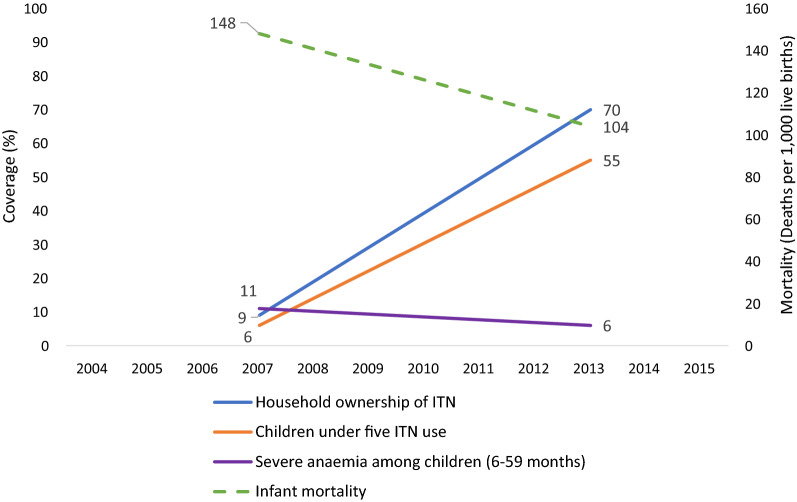
Summary of trends in intervention coverage, severe anaemia, and malaria mortality in children under five, in the DRC from 2007 to 2013. *ITN* insecticide-treated net

At the national level, the prevalence of severe anaemia in children decreased significantly, from 11% (95% CI 9–13%) in 2007 to 6% (95% CI 5–7%) in 2013. At the subnational level, a significant decrease in the prevalence of severe anaemia among children was observed in the provinces of Equateur, from 16% (95% CI 10–24%) in 2007 to 5% (95% CI 3–8%) in 2013, and Oriental, from 16% (95% CI 10–25%) in 2007 to 8% (95% CI 5–13%) in 2013 (Fig. 5). The national prevalence of severe anaemia compared with intervention coverage matches an increase in coverage of malaria intervention (Fig. 6).

#### Mortality: change in ACCM

**Fig. 7:**
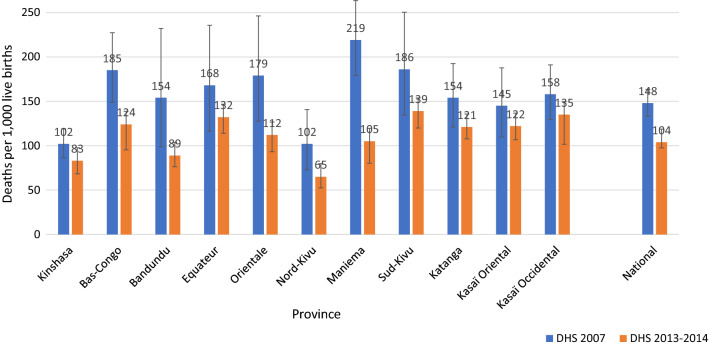
Trends in all-cause mortality among children under five by province, in the DRC from 2007 to 2013

The ACCM rate significantly declined between 2007 and 2013–2014 by 30%, from 148 (95% CI 133–163) per 1000 live births in 2007 to 104 (95% CI 98–111) per 1000 live births in 2013. By region, the largest decreases (relative reductions ranging from 15 to 52%) were found in Bas-Congo, Bandundu, Maniema, and Oriental provinces (Fig. 7). The observed decrease of ACCM is inversely proportional to the increase in ownership and use of ITNs (Fig. 6).

### Change in mortality risk associated with scale-up of ITN ownership

#### Kaplan–Meier survival probability

**Fig. 8:**
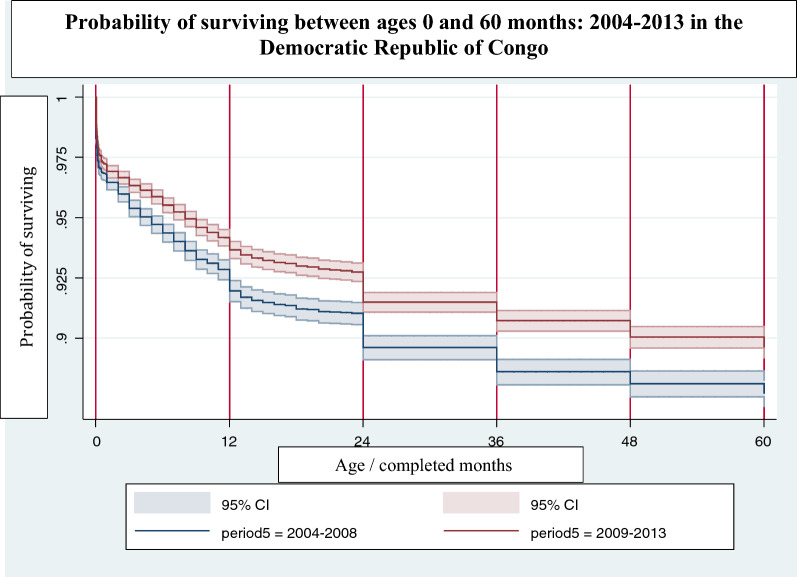
Kaplan–Meier survival probability. *CI* confidence interval

Kaplan–Meier survival probability analysis shows a significant improvement in survival probability of children aged 0–59 months between 2004 and 2013–2014. The survival probability was significantly higher among children born between 2009 and 2013, the period corresponding to the scale-up of malaria control interventions, compared to children born between 2004 and 2008, when the coverage of interventions was still low (Fig. 8). Among children aged 24–36 months, the probability of survival among children in 2004–2008 was approximately 89%, compared to 92% among children in 2009–2013.

#### Cox regression

The Cox proportional hazards regression examined the risk of mortality among children aged 0–59 months in relation to household ownership of ITNs. Household ownership of at least one ITN reduced the risk of mortality among children aged 0–59 months by 24 percentage points during the 24-month period before the survey (risk ratio = 0.76, 95% CI 0.64–0.90) (Table 2).

**Table 2:** Effect of household ITN ownership on 0–59-month mortality with the Cox regression model in DRC from 2011 to 2013

Variables	Risk ratio (95% CI)
Predictor	
Household ITN ownership	
Household without ITN	Reference
Household owning at least 1 ITN	**0.76 (0.64–0.90)**
Characteristics	
Age of the child (in months)	
< 6	Reference
6–11	**0.40 (0.31–0.51)**
12–23	**0.20 (0.14–0.27)**
24–35	**0.09 (0.06–0.15)**
36–47	**0.06 (0.03–0.11)**
47–59	**0.02 (0.01–0.08)**
Sex of the child	
Male	Reference
Female	0.97 (0.82–1.14)
Mother’s education	
No education	Reference
Primary	1.21 (0.97–1.5)
Secondary or higher	1.13 (0.87–1.46)
Mother’s marital status	
Single	Reference
Married	0.72 (0.46–1.11)
Single/widow	0.98 (0.60–1.60)
Household wealth quintiles	
Lowest	Reference
Second	0.98 (0.77–1.23)
Middle	1.02 (0.81–1.30)
Fourth	0.91 (0.69–1.20)
Highest	**0.45 (0.30–0.68)**
Place of residence	
Rural	Reference
Urban	**0.77 (0.61–0.96)**
Person month	367,405.1
N (death)	19,820 (550)

Bold = statistically significant

### Modest change in contextual factors

Trends in contextual factors such as socioeconomic, maternal and child health interventions, and meteorological factors, were examined [19, 20]. Gross domestic product per capita increased, from 218 USD in 2005 to 497 USD in 2015; however, the overall health expenditure also increased, from 9 USD in 2005 to more than 19 USD in 2015 [8, 21]. Most indicators of living conditions and sanitation were not significantly different from 2007 to 2013, such as access to improved water source (46% to 49%) and access to improved sanitation (15% to 18%) (Appendix). From 2007 to 2013, most indicators of maternal and child health in DRC did not significantly improve. Two indicators of antenatal care, which were relatively high in 2007, improved modestly in 2013, with one being statistically significant. The proportion of women who gave birth in a health facility increased, from 70.1% (95% CI 69.2–71.0%) in 2007 to 79.9% (95% CI 79.3–80.5%) in 2013. The proportion of births assisted by skilled personnel increased, from 74.0% (95% CI 70.0–78.0%) in 2007 to 80.1% (95% CI 77.6–82.7%) in 2013. The nutritional status among children under five assessed through the proportion receiving vitamin A also increased, from 54.6% (95% CI 50.4–58.8%) in 2007 to 70.3% (95% CI 67.9–72.6%) in 2013 and a decrease in stunting from 45.5% (95% CI 42.7–48.4) in 2007 to 42.4% (40.4–44.3%) in 2013. Of the child health indicators examined, only two have seen an increase greater than 10 percentage points between 2007 and 2013 [22]. Full basic vaccination increased, from 30.6% (95% CI 25.9–35.2%) in 2007 to 45.3% (95% CI 42.0–48.6%) in 2013, and exclusive breastfeeding increased, from 36.1% (95% CI 33.0–35.2%) in 2007 to 47.6 (95% CI 45.5–49.8%) in 2013.

In addition to these contextual factors, the total annual rainfall over the assessment period, calculated from the difference between the total annual precipitation for the year and total annual precipitation for the previous 5 years, has mostly deviated between − 50 and + 50 mm, except for 2006, which had higher excess rain of 188 mm [9]. The average temperatures also experienced positive and negative deviations between − 0.5 and 0.5 °C during the evaluation period. However, despite these differences, mean annual temperatures remained between 24 and 25 °C, which are favourable temperatures for malaria transmission. The total annual rainfall and temperature remained consistent and conducive to malaria transmission throughout the evaluation period; this regularity provides an appropriate background to evaluate the other indicators.

## Discussion

This study identified a significant decrease in severe anaemia and ACCM in DRC from 2007 to 2013–2014. This downward trend is most likely driven by household ownership of ITNs and use of ITNs among children under five. During this same period, there was a significant increase in financial investments by the country and its partners, focused on the scale-up of malaria control interventions, particularly vector control, from 18 million USD in 2007 to 175 million USD in 2015 [23, 24]. The NMCP implemented three ITN mass distribution campaigns across the country from 2009 to 2014, with a plan to distribute 1–3 ITNs per household or about 35 million ITNs total [25, 26]. This strategy and investments led to the significant increase in household ITN ownership and use among children under five. DRC nearly achieved its ITN ownership coverage target of 80%. ITN ownership and use targets are often difficult to achieve. In a study of 22 sub-Saharan African countries, only 4 achieved their household ITN ownership targets, and none achieved their ITN use among children under five targets [27].

The results show a clear pattern, which can be observed through the changes across the 11 provinces. The subnational analysis revealed an overall increase in ITN ownership and a significant decrease in ACCM and severe anaemia, but most importantly, some of the provinces with the largest improvements in coverage of ITN ownership and use also reported the largest decrease in ACCM. A finding reflected in a similar assessment showed a strong correlation between a high level of ITN use and the protective effect against malaria among children under five in DRC [28]. The geospatial differences in provinces may explain why some provinces with high intervention coverage experienced minimal change in either severe anaemia or ACCM. These spatial differences result in varying levels of access to health care, implementation of specific health programmes, and exposure to conflict, which may affect health outcomes of children under five [29]. Families fleeing areas of significant conflict in east DRC may have affected ACCM in those areas and contributed to the slight decreases in ACCM observed in neighbouring provinces such as Equateur, Katanga, and Sud-Kivu. Provinces without conflict and high intervention coverage, such as Bandundu, experienced the second largest decrease in ACCM.

The implementation of routine distribution of ITNs through antenatal clinics and EPI services, with a focus on younger children, may have resulted in the more pronounced decline in child mortality among children aged 6–23 months, compared to those aged 24–59 months. This is significant because children aged 6–23 months are more susceptible to malaria mortality [30]. Similar results were found by Dolan et al., who identified a 41% reduction in ACCM among children living in rural areas due to ITN distribution campaigns that targeted the highest risk areas for malaria in DRC [31]. Furthermore, this observation is consistent with other studies in sub-Saharan Africa that found an association between an increase in ITN ownership and a decline in ACCM and severe anaemia [32–34].

The results of the Kaplan–Meier survival probability analysis and Cox proportional hazards regressions both indicated an improved probability of survival among children under five post-intervention scale-up, compared to pre-intervention scale-up, which further supports the association between the increase in ITN coverage and decrease in ACCM. These methods have been used in similar studies assessing ITN effectiveness in reducing child mortality [35].

In addition to the scale-up of ITNs, the NMCP planned to implement several interventions during the evaluation period, however, the results were not as promising. The trends analysis revealed that access to ACT was still very low. In addition, low adherence to treatment policies and use of non-ACT were prevalent across the country [36]. Low access to ACT may have contributed to the increase in severe anaemia in Sud-Kivu, and minimal changes in Katanga and Bandundu, despite high ITN ownership and use. The deployment of IRS across the country has been limited to one province due to funding, technical and logistical challenges [25]. The scale-up of IPTp across the country has not reached a comparable scale to ITNs [25]. A study conducted in 2013 found that only 20% of public health facilities in DRC were stocking sulfadoxine-pyrimethamine for IPTp [37]. The unmet goals for these interventions strengthen the plausible attribution of ITN ownership and ITN use to the observed decrease in ACCM.

To further investigate potential factors that may have contributed to the decline in ACCM, the contextual factor analysis indicated only a modest improvement in living conditions, sanitation, and maternal and child health indicators, such as nutritional status, vaccination status, exclusive breastfeeding, and access to water and improved toilets from 2007 to 2014 [2, 38, 39]. There were also no substantial changes in climate, rainfall, and temperature between 2005 and 2014 that may have affected the national mortality trend. Based on the analysis of these key factors known to affect malaria incidence, it is unlikely that these non-malaria contextual factors can explain a large proportion of the 30% reduction of ACCM [40, 41].

Overall, these results support the conclusion that the increase in household ITN ownership and increase in ITN use among children under five have contributed to the observed decrease in ACCM and severe anaemia from 2005 to 2014 in DRC. This study provides an assessment of the intervention coverage achieved and describes malaria-related morbidity and mortality rates up to 2014 in DRC. These results also provide baseline data that will be important for the measurement of the imminent progress that will be achieved at the end of the current 2016–2020 NMSP and may inform the decision-making of the NMCP and its partners as they transition to a new NMSP. Further research will be needed to fully understand how an increase in ITN ownership and use results in decreased ACCM and severe anaemia. However, DRC has made significant progress since 2005 in increasing intervention coverage and decreasing the burden of malaria on the population.

## Limitations of the study

The use of secondary data from the DHS 2007 and DHS 2013–2014 limited this study to the variables that were collected in those surveys. No further primary data were collected. The difference in methodology between national surveys precluded the inclusion of the 2010 and 2017–2018 DRC Multiple Indicator Cluster Survey, which may have provided another data point to help elucidate the effects of DRC’s malaria control intervention scale-up. Without data on malaria parasite prevalence from DHS 2007, the authors could not assess the parasitaemia trends. Other factors which can potentially affect ACCM, such as spatial differences in health programme implementation, climate differences across provinces, ongoing conflict, and scale-up of other disease interventions should be accounted for in future analysis [29, 42].

## Conclusion

DRC has made significant gains in the coverage of and access to key malaria control interventions, such as ITNs. As DRC continues to focus its energy on scaling up malaria control strategies, the results are encouraging for future gains against the disease. The evidence in this study supports the conclusion that the scale-up of malaria control interventions substantially contributed to the observed decline in ACCM in DRC from 2005 to 2014, despite the high malaria prevalence in the country. When the next DHS survey is conducted in DRC, a similar analysis will be helpful to evaluate the achievements and impact of DRC’s 2016–2020 NMSP.

## Data Availability

The data that support the findings of this study are available from The DHS Program upon reasonable request and with permission of The DHS Program.

## Appendix

See Tables 3, 4 and 5

**Table 3:** Changes in contextual factors associated with household factors in DRC from 2007 to 2014. Source: DHS 2007 and 2013–2014

Characteristics	2007 DHS		2013–2014 DHS		Absolute change 2014–2007
	(95% CI)	N	(95% CI)	N	
Household characteristics					
Access to safe drinking water	46.2 (39.9–52.7)	8886	48.7 (44.2–53.3)	18,171	2.5
Access to improved toilets	15.6 (12.9–19.6)	8886	18.4 (16.1–21.0)	18,171	2.8
Household with flooring	19.5 (16.1–23.3)	8886	17.6 (15.2–20.3)	18,171	1.8
Household with electricity	15.3 (12.3–18.8)	8886	13.5 (11.1–16.4)	18,171	1.8
Household with telephone (fixed or mobile)	**31.3 (30.3–32.3)**	**8886**	**39.9 (39.2–40.6)**	**18,171**	**8.6**
Socio-demographic characteristics					
Proportion of women aged 15–49 who have at least reached primary education	**79.2 (75.6–82.3)**	**9995**	**84.6 (82.6–86.3)**	**18,827**	**5.4**
Proportion of married 15–49 year old women (in union)	66.3 (64.1–68.3)	9995	64.2 (62.5–66.0)	18,827	2.1

Drinking water sources: tap, fountain, borehole, protected wells, rainwater, and bottled water. Toilets not shared by other households: flush toilet, improved latrine, latrine with slab, composting, according to DHS standards. Floor covering includes cement, tile, carpet, other modern material

Bold = statistically significant

*CI* confidence interval, *DHS* demographic and health survey

**Table 4:** Changes in contextual factors associated with maternal health in DRC from 2007 to 2014. Source: DHS 2007 and 2013–2014

Characteristics	2007 DHS		2013–2014 DHS		Absolute change 2014–2007
	(95% CI)	N	(95% CI)	N	
Fertility risks					
Single high-risk birth^a^	**36.4 (35.4–37.4)**	**9003**	**38.8 (38.1–39.5)**	**18,390**	**2.4**
High-risk multiple-risk birth	25.0 (24.1–25.9)	9003	24.8 (24.2–25.4)	18,390	0.2
Avoidable risk birth^b^	**61.3 (60.3–62.3)**	**9003**	**63.6 (62.9–64.3)**	**18,390**	**2.3**
Unavoidable risk birth^c^	15.9 (14.5–16.7)	9003	14.1 (13.6–14.6)	18,390	1.8
Birth at intervals—24 months	7.3 (6.5–7.9)	9003	8.2 (7.5–8.8)	18,390	0.9
Birth order > 3	22.8 (21.9–23.7)	9003	23.9 (23.3–24.5)	18,390	1.1
Mother of age < 18 and > 34 years old	6.3 (5.8–6.8)	9003	6.7 (6.3–7.1)	18,390	0.4
Coverage in prenatal care					
Prenatal consultation (ANC)	46.7 (45.4–48.0)	5473	48.0 (47.1–48.9)	11,065	1.3
At least 2 TT doses during pregnancy	**38.6 (35.6–41.5)**	**5473**	**43.2 (42.3–44.1)**	**11,065**	**4.6**
Childbirth in a health facility^d^	**70.1 (69.2–71.0)**	**8999**	**79.9 (79.3–80.5)**	**18,390**	**9.8**
Birth assisted by qualified staff	**74.0 (70.0–78.0)**	**8999**	**80.1 (77.6–82.7)**	**18,390**	**6.1**

Bold = statistically significant

*TT* Tetanus toxoid vaccine, *CI *confidence interval, *DHS* demographic and health survey

^a^High-risk birth is defined as a birth that occurred before 24 months of the previous birth, multiple delivery, birth order < 3, or a mother’s age is < 18 years old or > 34 years old

^b^Avoidable risk birth is defined as a birth at an age of < 18 years old or > 34 years old, interval of < 24 months between births or a birth order > 3

^c^Unavoidable risk birth is defined as any childbirth < 18 or > 34 years old

^d^Concerns childbirth in all public semi-public and private structures

**Table 5:** Changes in contextual factors associated with child survival in DRC from 2007 to 2014. Source: DHS 2007 and 2013–2014

Characteristics	2007 DHS		2013–2014 DHS		Absolute change 2014–2007
	(95% CI)	N	(95% CI)	N	
Vaccine coverage					
BCG	**71.7 (70.3–72.3)**	**1632**	**83.5 (81.1–85.7)**	**3634**	**11.8**
DTP3	**45.0 (43.9–46.2)**	**1632**	**61.0 (57.4–64.6)**	**3634**	**16.0**
Polio3	**45.7 (43.5–48.4)**	**1632**	**65.5 (62.2–68.6)**	**3634**	**19.8**
Measles	**62.9 (60.5–65.2)**	**1632**	**72.2 (69.7–74.7)**	**3634**	**9.3**
Total vaccine coverage	**30.6 (25.9–35.2)**	**1585**	**45.3 (42.0–48.6)**	**3634**	**14.7**
Micronutrient supplementation and nutritional status					
Vitamin A	**54.6 (50.4–58.8)**	**1585**	**70.3 (67.9–72.6)**	**15,271**	**15.7**
Delayed growth	45.5 (42.7–48.4)	3631	42.4 (40.4–44.3)	8059	− 3.1
Underweight	25.1 (22.2–27.9)	3631	22.4 (20.8–24.0)	8059	− 2.7
Emaciation	10 (8.0–12.1)	3631	7.9 (7.0–9.0)	8059	− 2.1
Low birth weight (< 2500 g)	7.7 (6.9–8.4)	6104	7.1 (6.3–7.8)	13,922	0.6
Early initiation (≤ 1 h of childbirth)	**48.1 (46.7–49.4)**	**5266**	**51.9 (50.7–53.1)**	**7168**	**3.8**
Maternal breastfeeding					
Exclusive breastfeeding (6 months)	**36.1 (33.0–39.2)**	**927**	**47.6 (45.4–49.8)**	**1935**	**11.5**
% of 6–9 months of breastfeeding and complementary food	82.2 (79.0–85.4)	555	78.7 (75.5–81.0)	1209	3.5
Coverage of integrated management of ‘child’s disease’					
Diarrhea rehydration therapy	44.9 (42.2–47.6)	1287	41.9 (38.9–44.9)	2818	3.0
Oral rehydration salts for diarrhea	**30.8 (28.2–33.3)**	**1287**	**39.2 (36.4–42.2)**	**2818**	**8.3**
Other childhood diseases					
Prevalence of diarrhea	16.4 (15.6–17.2)	7987	16.8 (15.6–18.0)	17,017	0.4
Prevalence of ARI symptoms	**15.4 (14.6–16.2)**	**7987**	**6.7 (5.9–7.1)**	**17,017**	**8.7**

Percentage of children aged 12–23 months with recommended vaccinations [this coverage takes into account vaccines against BCG (against tuberculosis), three doses of diphtheria, tetanus, pertussis (DTP3), three doses of oral polio vaccine (Polio3), and measles]; after 2007 the DTP vaccine were given pentavalent form with antigens against Hepatitis B and *Haemophilus influenzae* type B included

Bold = statistically significant

*ARI* acute respiratory infections, *N* weighted sample, *CI* confidence interval, *DHS* demographic and health survey

## Abbreviations

ACCM: All-cause child mortality
ACT: Artemisinin-based combination therapy
CI: Confidence interval
EPI: Expanded programme on immunization
DHS: Demographic and Health Survey
DRC: Democratic Republic of Congo
IPTp: Intermittent preventive treatment in pregnancy
ITN: Insecticide-treated net
NMCP: National Malaria Control Programme
NMSP: National malaria strategic plan
